# Real-world outcomes of ustekinumab, vedolizumab, and tumor necrosis factor inhibitors in very-early-onset inflammatory bowel disease: a multi-center cohort study

**DOI:** 10.1007/s00535-025-02334-9

**Published:** 2026-01-03

**Authors:** Ryusuke Nambu, Itaru Iwama, Ichiro Takeuchi, Shin-ichiro Hagiwara, Yuri Etani, Emiri Kaji, Atsushi Yoden, Fumihiko Kakuta, Yusuke Hoshi, Naoya Tsumura, Tatsuki Mizuochi, Hideki Kumagai, Koji Yokoyama, Takuya Nishizawa, Masaaki Usami, Yugo Takaki, Ryo Ebana, Shingo Kurasawa, Hiroki Fujikawa, Takashi Ishige, Takahiro Kudo, M. Masashi Yoshida, Hirotaka Shimizu, Katsuhiro Arai

**Affiliations:** 1 Center for Pediatric Inflammatory Bowel Disease, Division of Gastroenterology and Hepatology, Saitama Children’s Medical Center, 1-2 Shintoshin, Chuo-Ku, Saitama, 3308777 Japan; 2 Center for Pediatric Inflammatory Bowel Disease, Division of Gastroenterology, National Center for Child Health and Development, Tokyo, Japan; 3 Department of Gastroenterology, Nutrition and Endocrinology, Osaka Women’s and Children’s Hospital, Osaka, Japan; 4 Department of Pediatrics, Osaka Medical and Pharmaceutical University, Osaka, Japan; 5 Department of Pediatrics, Kawanishi City Medical Center, Hyogo, Japan; 6 Department of Gastroenterology, Miyagi Children’s Hospital, Miyagi, Japan; 7 Department of Pediatrics and Child Health, Kurume University School of Medicine, Fukuoka, Japan; 8 Department of Pediatrics, Jichi Medical University, Tochigi, Japan; 9 Department of Pediatrics, Gunma University Graduate School of Medicine, Gunma, Japan; 10 Department of Pediatrics School of Medicine, Institute of Medical, Pharmaceutical and Health Sciences, Kanazawa University, Ishikawa, Japan; 11 Department of Pediatric Gastroenterology and Hepatology, Japanese Red Cross Kumamoto Hospital, Kumamoto, Japan; 12 Department of Pediatrics, Okinawa Chubu Hospital, Okinawa, Japan; 13 Department of Pediatrics, Shinshu University School of Medicine, Nagano, Japan; 14 Department of Pediatrics, Hiroshima University Graduate School of Biomedical and Health Sciences, Hiroshima, Japan; 15 Department of Pediatrics, Faculty of Medicine, Juntendo University, Tokyo, Japan

**Keywords:** Very-early-onset IBD, Pediatric inflammatory bowel disease, Biologics

## Abstract

**Background:**

Very-early-onset inflammatory bowel disease (VEO-IBD), representing cases diagnosed before age 6 years, is increasing in prevalence. Although VEO-IBD often presents as severe, treatment-resistant disease requiring biologic agents, studies showing the effectiveness of biologics, such as ustekinumab (UST) and vedolizumab (VDZ), remain limited.

**Methods:**

We retrospectively analyzed patients with VEO-IBD treated for at least a year from 13 institutions in Japan, evaluating clinical course including effectiveness of biologics, such as infliximab (IFX), adalimumab (ADL), UST, and VDZ. Patients with monogenic IBD were excluded. Steroid-free clinical remission (SFCR) and treatment persistence were assessed separately for first-line and for second-line or subsequent biologic therapies.

**Results:**

We studied 101 VEO-IBD patients (56% male; median age, 3.6 years), including 40 with Crohn’s disease, 52 with ulcerative colitis, and 9 with unclassified IBD. Biologics were used in 67 patients, most commonly infliximab (IFX; *n* = 52), followed by UST (*n* = 38), adalimumab (ADL; *n* = 23), and VDZ (*n* = 21). As first-line therapy, IFX and ADL achieved 1-year SFCR rates of 19% and 46%, with persistence rates of 36% and 48%. Despite being used mainly as second-line or subsequent therapies, UST and VDZ showed 1-year SFCR rates of about 45% and 36%, and maintained persistence of 79% and 46%, respectively, with UST demonstrating higher persistence than TNF-α inhibitors (*P* < 0.01). No discontinuations due to infusion reactions or other adverse events occurred with UST or VDZ.

**Conclusion:**

UST and VDZ were effective and well tolerated even when used as second-line or subsequent therapies for VEO-IBD.

**Supplementary Information:**

The online version contains supplementary material available at 10.1007/s00535-025-02334-9.

## Introduction

Inflammatory bowel disease (IBD), a group of chronic, relapsing intestinal immune disorders, includes ulcerative colitis (UC), Crohn’s disease (CD), and IBD-unclassified (IBDU). Incidence of pediatric IBD has risen worldwide, accompanied by younger average age at diagnosis. Incidence of very-early-onset IBD (VEO-IBD), diagnosis before age 6 years, has increased particularly rapidly [1, 2].

VEO-IBD can be classified into monogenic IBD, caused by single-gene mutations and accounting for approximately 10–15% of cases, and non-monogenic IBD, the remaining 85–90% [3]. In monogenic IBD, treatment is tailored to the underlying disease mechanism. Non-monogenic VEO-IBD is typically managed with treatment strategies resembling those for older children and adults, but even non-monogenic VEO-IBD appears strongly influenced by genetic predisposition and often is characterized by severe disease, treatment resistance, perianal involvement, and significant growth impairment. Biologic therapies frequently prove necessary [4–6]. Rates of biologic use in pediatric IBD have increased, with reports indicating use in 43 to 64% of general pediatric IBD populations [7–9]. Comparative data regarding efficacy, safety, and real-world use of biologics in VEO-IBD remain limited. Tumor necrosis factor-alpha (TNF) inhibitors, such as infliximab (IFX) and adalimumab (ADL), have been approved for pediatric use and are increasingly reported in this population [10, 11]. However, agents awaiting pediatric approval, such as ustekinumab (UST) and vedolizumab (VDZ), are less studied in real-world settings.

To guide treatment strategies for VEO-IBD following the anticipated pediatric approval of UST and VDZ, we conducted a multicenter real-world study using data collected from IBD centers across Japan.

## Methods

### Study design

This retrospective cohort study was conducted at 13 pediatric centers in Japan specializing in IBD. Eligible participants were diagnosed with VEO-IBD before age 6 between April 1, 2017 and September 30, 2023, with at least 1 year of follow-up. All participating institutions had certified pediatric gastroenterologists accredited by the Japanese Society for Pediatric Gastroenterology, Hepatology and Nutrition who were members of the Japanese Pediatric IBD Research Group. Our study aimed to characterize the natural history of VEO-IBD and retrospectively analyze clinical course in patients given biologic agents, such as IFX, ADL, UST, and VDZ. Patients whose genetic testing identified monogenic IBD were excluded, considering their distinct characteristics and treatment responses [12].

### Outcomes

Primary outcomes studied were achievement of steroid-free clinical remission (SFCR) and persistence of remission at 6 and 12 months after initiation of each biologic agent. These outcomes were analyzed separately for biologics used as first-line therapy and for those used as second-line or subsequent therapy, focusing on time to discontinuation as a measure of persistence. In addition, factors associated with early discontinuation of each biologic were explored. Important secondary outcomes included reasons for discontinuation of biologics; and, among patients given UST and VDZ (both unapproved for pediatric use), comparison between continuation and discontinuation groups of weight-based dose at initiation and final dose at discontinuation or the end of follow-up.

### Data collection

Data were extracted from medical records at times of diagnosis, initiation of each biologic therapy, and every 6 months (± 3 weeks) thereafter until the observation period ended. For non-biologic medications, dates of initiation and discontinuation were recorded for each agent. Using a predesigned data collection form, the following information was obtained: baseline patient characteristics at study entry, disease type, treatment history before biologic initiation, dosing and frequency of biologic therapies, clinical scores (Pediatric Ulcerative Colitis Activity Index (PUCAI) or weighted Pediatric Crohn’s Disease Activity Index (wPCDAI)), and standard laboratory parameters (hemoglobin, albumin, C-reactive protein, and erythrocyte sedimentation rate). In addition, surgical procedures, such as intestinal resection, stoma creation, and seton insertion, during the observation period were recorded. Data concerning final dose and interval were excluded from analysis if the biologic agent was discontinued (or follow-up ended) within 2 months of initiation. If the same biologic agent was used more than once in the same patient after a treatment-free interval, only the data from the initial course were included in the analysis. Variables with less than 3% missing data were included; patients with missing values for a specific variable were excluded from that analysis (pairwise deletion).

### Definitions

Diagnoses were made by certified pediatric gastroenterologists at each participating center in accord with the revised Porto criteria [13]. Disease phenotypes for CD and UC/IBDU were assigned according to the Paris classification [14]. Clinical disease activity was assessed using wPCDAI for CD and PUCAI for UC/IBDU [15, 16]. Severe disease was defined as a PUCAI score ≥ 65 or a wPCDAI score > 57.5. Clinical remission was defined as a PUCAI score < 10 or a wPCDAI score < 12.5. SFCR was defined as clinical remission in the absence of systemic corticosteroid use at the corresponding time point. Persistence was defined as continued use of the biologic agent without discontinuation or changing to another biologic. Among reasons for discontinuation, secondary loss of response was defined as loss of efficacy after achieving clinical remission, while primary non-response was defined as failure to achieve remission at any time point throughout administration.

### Statistical analysis

Categorical variables were presented as frequencies and percentages, while continuous variables were expressed as medians with interquartile ranges (IQR). Patient characteristics were compared among four biologic agents (IFX, ADL, UST, and VDZ) used as second-line or subsequent therapies using Fisher’s exact test for categorical variables and the Kruskal–Wallis test for continuous variables. SFCR rates at 6 and 12 months after initiation of each biologic were descriptively summarized, and 95% confidence intervals were calculated using the Clopper–Pearson exact method. Two patients who discontinued therapy within 3 months at their request were excluded from the 6-month SFCR analysis as they had not reached the assessment time point. Persistence rate was analyzed using the Kaplan–Meier method, and group comparisons were performed using the log-rank (Mantel–Cox) test. Comparisons were conducted among biologics used as second-line or subsequent therapies. To explore factors associated with early discontinuation of biologic therapy, univariable analysis was performed using the log-rank test, as well as multivariable analysis using the Cox proportional hazards model. Multivariable analysis was limited to IFX because of the small number of discontinuation events in other biologics. For UST and VDZ, weight-based dosing at initiation and final administration was compared between ongoing treatment and discontinuation groups using the Mann–Whitney *U* test. All analyses were two-sided, and *P* values < 0.05 were considered statistically significant. Statistical analysis was conducted using GraphPad Prism (version 10.4.0, GraphPad Software, San Diego, CA, USA).

### Ethical considerations

This study was conducted in accord with the Declaration of Helsinki (2013 revision) and approved by the central ethics committee of Saitama Children's Medical Center (approval number: 2024-04-016) and by the institutional review boards of all participating centers. Informed consent was obtained using an opt-out approach via institutional websites.

## Results

### Characteristics and natural history

Among 101 patients included, 56 (56%) were male. Median age at diagnosis was 3.6 years (IQR: 2.6–5.3). Disease entities included CD in 40 patients, UC in 52, and IBD-U in 9 (Table 1). Genetic testing was performed in 78 patients, and no known monogenic IBD was identified.

**Table 1:** Baseline patient characteristics

	*n* = 101
Male, *n*	56
Age at diagnosis, median (IQR), years	3.6 (2.6–5.3)
Phenotype, *n*	
CD	40
UC	52
IBD-U	9
Family history^#^, *n*	6
Observation period, median (IQR), years	3.8 (2.4–5.0)
Severe disease at diagnosis, *n*	
CD, wPCDAI > 57.5, *n* = 40	13
UC/IBDU, PUCAI ≥ 65, *n* = 61	10
Laboratory data, median (IQR)	
Hgb (mg/dL)	11.0 (9.4–11.9)
Alb (g/dL)	3.5 (2.9–4.2)
CRP (mg/dL)	0.31 (0.08–1.16)
ESR (mm/1 h)	20 (10–38)
Non-biologic medication, *n*	
5-ASA	79
Systemic corticosteroid	71
IM	66
Tac/Cys	7
Number of biologic agents, *n*	
≥ 1	67
≥ 2	45
≥ 3	23
Biologic agents used, *n*	
IFX	52
UST	38
ADL	23
VDZ	21
TOF	3
UPA	3
RKZ	3
GLM	2
MKZ	1
Surgery, *n*	
Colectomy	6
Ileostomy/colostomy	7
Seton insertion	6

*IQR* interquartile range, *CD* Crohn’s disease, *UC* ulcerative colitis, *IBD-U* inflammatory bowel disease-unclassified, *wPCDAI* weighted pediatric Crohn’s disease activity index, *PUCAI* pediatric ulcerative colitis activity index, *Hgb* hemoglobin, *ALB* albumin, *CRP* C-reactive protein, *ESR* erythrocyte sedimentation rate, *ASA* aminosalicylic acid, *IM* immunomodulators, *TAC/Cys* tacrolimus/cyclosporine, *IFX* infliximab, *UST* ustekinumab, *ADL* adalimumab, *VDZ* vedolizumab, *TOF* tofacitinib, *UPA* upadacitinib, *RKZ* risankizumab, *GLM* golimumab, *MKZ* mirikizumab

^#^Within second-degree relatives

Among patients with CD, the most common disease location was colonic (L2), observed in 60%; 90% of all patients had some colonic involvement. Strictures and perforations were rare, occurring in 3 and 1 patients, respectively. Perianal disease was present in 18 of 40 CD patients (45%). In the UC/IBD-U group, 89% had pancolitis (E4) and 10 patients (16%) met criteria for severe disease at diagnosis (Table 1 and Supplementary Table 1).

**Fig. 1:**
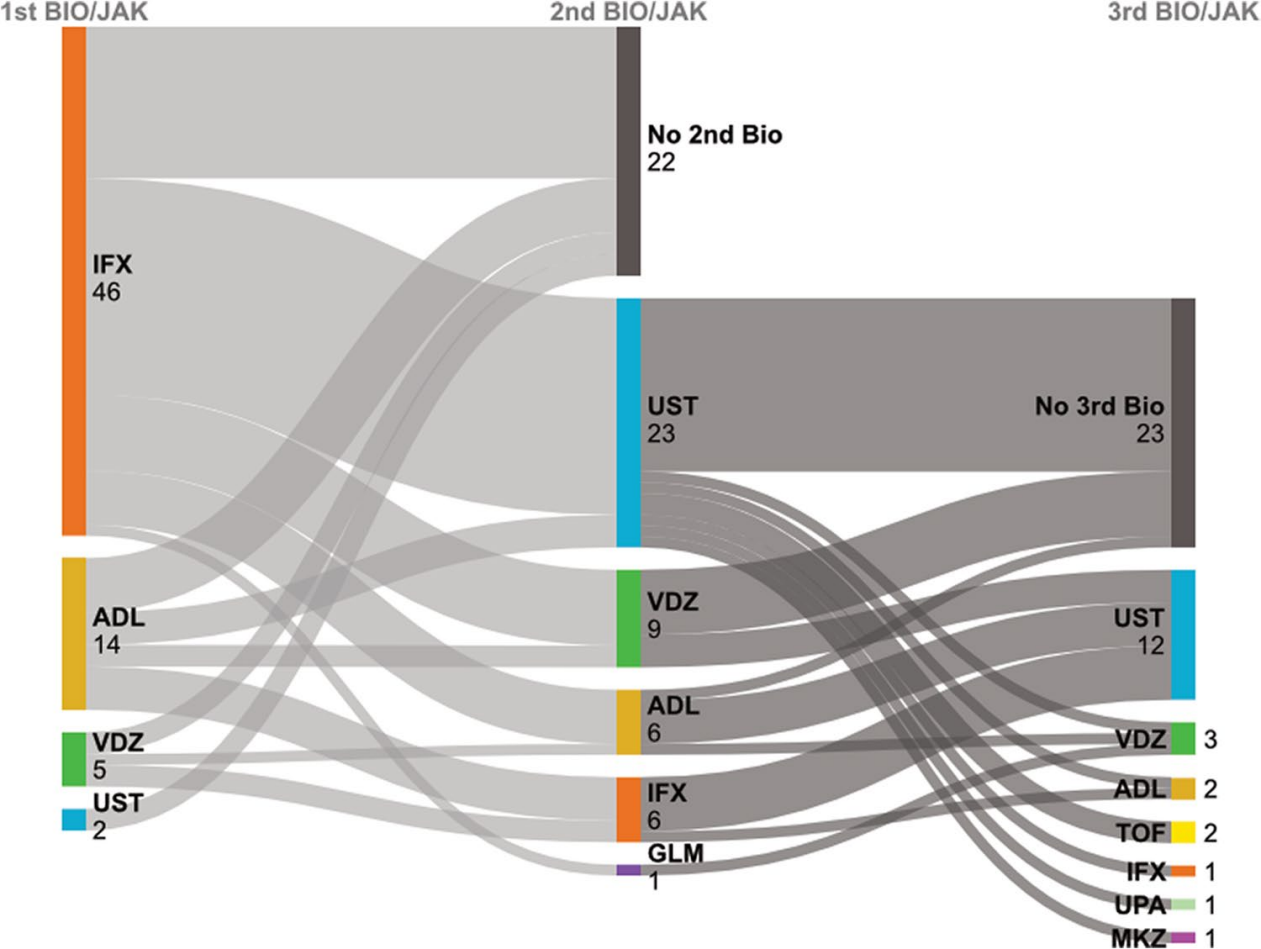
Treatment sequences of biologic and JAK inhibitor therapies shown as a Sankey diagram. The figure shows sequences of therapies used in individual patients, from first-choice to third-line treatments. Width of each stream represents numbers of patients switched from one agent to another across lines of therapy. *BIO* biologics, *JAK* Janus kinase inhibitor, *IFX* infliximab, *ADL* adalimumab, *VDZ* vedolizumab, *UST* ustekinumab, *GLM* golimumab, *TOF* tofacitinib, *UPA* upadacitinib, *MKZ* mirikizumab

Biologic therapies were used in 67 patients (67%). The most frequently used agent was IFX (*n* = 52), followed by UST (*n* = 38), ADL (*n* = 23), and VDZ (*n* = 21). During the observation period, 45 patients required a second biologic while 23 progressed to a third or subsequent agent. Bowel resection was performed for 6 patients (4 with UC, 1 CD, and 1 IBD-U). Seton insertion was performed in 6 CD patients. Among patients who received biologic therapy, 90% were initially treated with a TNF inhibitor, with IFX used in 46 and ADL in 14 (Fig. 1 and Table 2a). UST was initiated as a second-line or subsequent therapy in 95% of its recipients (36/38), similarly to VDZ in 76% (16/21). Among patients receiving biologics as second-line or subsequent therapy, no significant differences were observed among agents regarding age at diagnosis, disease severity at initiation, or laboratory values at the start of each biologic, except for serum albumin, which was higher in the VDZ group (*P* = 0.04) (Table 2b).

**Table 2:** Baseline characteristics and treatment history of patients receiving each biologic as (A) first-line therapy and (B) second-line or subsequent therapy

A. First-line therapy				
	IFX (*n* = 46)	ADL (*n* = 14)	VDZ (*n* = 5)	UST (*n* = 2)
Sex, male, *n* (%)	20 (46)	12 (86)	4 (80)	1 (50)
Age at diagnosis, median (IQR)	3.5 (2.4–4.4)	5.0 (3.8–5.6)	4.3 (1.7–4.5)	4.4 (3.9–4.9)
Disease phenotype, *n* (%)				
CD	22 (48)	8 (57)	1 (20)	0 (0)
UC/IBDU	24 (52)	6 (43)	4 (80)	2 (100)
Family history of IBD^#^, *n* (%)	2 (4.3)	2 (14)	0 (0)	0 (0)
Observation period, median (IQR), years	3.0 (2.4–4.5)	4.3 (2.7–5.1)	3.0 (2.9–3.2)	3.6 (2.7–4.5)
Duration from diagnosis to initiation of each biologic, median (IQR), months	3.0 (1.0–10.8)	1.0 (0.3–14.3)	6.0 (4.0–8.0)	18.5 (12.8–24.3)
Perianal disease at the diagnosis, *n* (%*)	11(50*)	4 (50*)	1 (100*)	0 (0*)
Disease severity per Colitis Activity Index (wPCDAI > 57.5/PUCAI ≥ 65) at initiation of each biologic, *n* (%)	13 (28)	1 (7.1)	0 (0)	0 (0)
Laboratory values at initiation of each biologic, median (IQR)				
Hgb (g/dl)	11.1 (10–11.9)	11.3 (11.0–12.3)	9.1 (8.9–9.9)	11.8 (11.3–12.3)
Alb (g/dl)	3.7 (3.2–4.1)	3.6 (3.2–3.9)	3.6 (2.9–4.0)	4.2 (4.1–4.4)
CRP (mg/dl)	0.15 (0.04–0.57)	0.26 (0.04–0.76)	0.04 (0.02–0.16)	0.41 (0.22–0.59)
Treatment prior to each biologics, *n* (%)				
5-ASA	33 (72)	9 (64)	4 (80)	1 (50)
EEN^§^	20 (43)	7 (50)	1 (20)	1 (50)
Systemic corticosteroid	32 (70)	8 (57)	4 (80)	2 (100)
IM	34 (74)	6 (43)	1 (20)	1 (50)
Tac/Cys	3 (6.5)	1 (7.1)	0 (0)	0 (0)

B. Second-line or subsequent therapy					
	UST (*n* = 36)	VDZ (*n* = 16)	ADL (*n* = 9)	IFX (*n* = 6)	*P*
Sex, male, *n* (%)	17 (45)	11 (69)	4 (44)	6 (100)	0.05
Age at diagnosis, median (IQR)	3.7 (2.6–5.3)	3.5 (2.8–5.4)	1.9 (1.7–3.4)	5.5 (4.6–5.6)	0.41
Disease phenotype, *n* (%)					
CD	18 (50)	3 (19)	4 (44)	4 (67)	0.11
UC	18 (50)	13 (81)	5 (55)	2 (33)	
Family history of IBD, *n* (%)	3 (8.3)	2 (13)	1 (11)	1 (17)	0.81
Observation period, median (IQR), years	3.8 (2.5–4.9)	3.7 (2.6–4.9)	2.9 (2.3–3.8)	4.2 (3.1–5.5)	0.67
Duration from diagnosis to initiation of each biologic, median (IQR), months	12.0 (4.5–24.0)	21 (12.3–28.0)	9.0 (7.0–20.0)	7.5 (4.8–17)	0.09
Perianal disease at the diagnosis, *n* (%*)	7 (39*)	1 (33*)	2 (50*)	2 (50*)	1
Disease severity per Colitis Activity Index (wPCDAI > 57.5/PUCAI ≥ 65) at initiation of each biologic, *n* (%)	7 (19)	4 (25)	0 (0)	3 (50)	0.12
Laboratory values at initiation of each biologic, median (IQR)					
Hgb (g/dl)	11.2 (9.6–11.9)	10.4 (10.1–11.1)	11.3 (9.5–12.3)	11.4 (8.7–11.6)	0.62
Alb (g/dl)	3.4 (2.9–3.7)	3.8 (3.4–4.2)	3.3 (3.3–4.0)	3.2 (2.3–3.2)	0.04
CRP (mg/dl)	0.26 (0.08–0.90)	0.14 (0.04–1.8)	0.15 (0.05–0.98)	1.28 (0.88–2.04)	0.23
Treatment prior to each biologics, *n* (%)					
5-ASA	26 (72)	15 (94)	5 (56)	3 (50)	0.09
EEN^§^	20 (56)	3 (23)	5 (56)	2 (33)	0.07
Systemic corticosteroid	31 (86)	15 (94)	8 (89)	5 (83)	0.88
IM	29 (81)	14 (88)	6 (67)	2 (33)	0.05
Tac/Cys	3 (8.3)	5 (31)	1 (11)	0 (0)	0.14
Biologic treatment line, *n* (%)					
Second-line therapy	23 (64)	8 (50)	6 (67)	6 (100)	0.20
Third-line or later therapy	13 (36)	8 (50)	3 (33)	0 (0)	

*IFX* infliximab; *ADL* adalimumab; *VDZ* vedolizumab; *UST* ustekinumab; *IQR* interquartile range; *CD* Crohn’s disease; *UC* ulcerative colitis; *IBD-U* inflammatory bowel disease-unclassified; *wPCDAI* weighted pediatric Crohn’s disease activity Index; *PUCAI* pediatric ulcerative colitis activity index; *Hgb* hemoglobin; *Alb* albumin; *CRP* C-reactive protein; *5-ASA* 5-aminosalicylic acid; *IM* immunomodulators; *TAC/Cys* tacrolimus/cyclosporine

^#^Within second-degree relatives

*Percentages were calculated based on cases of Crohn’s disease

^§^EEN was defined as exclusive enteral nutrition maintained for ≥ 2 consecutive weeks

### Steroid-free clinical remission and treatment persistence

**Fig. 2:**
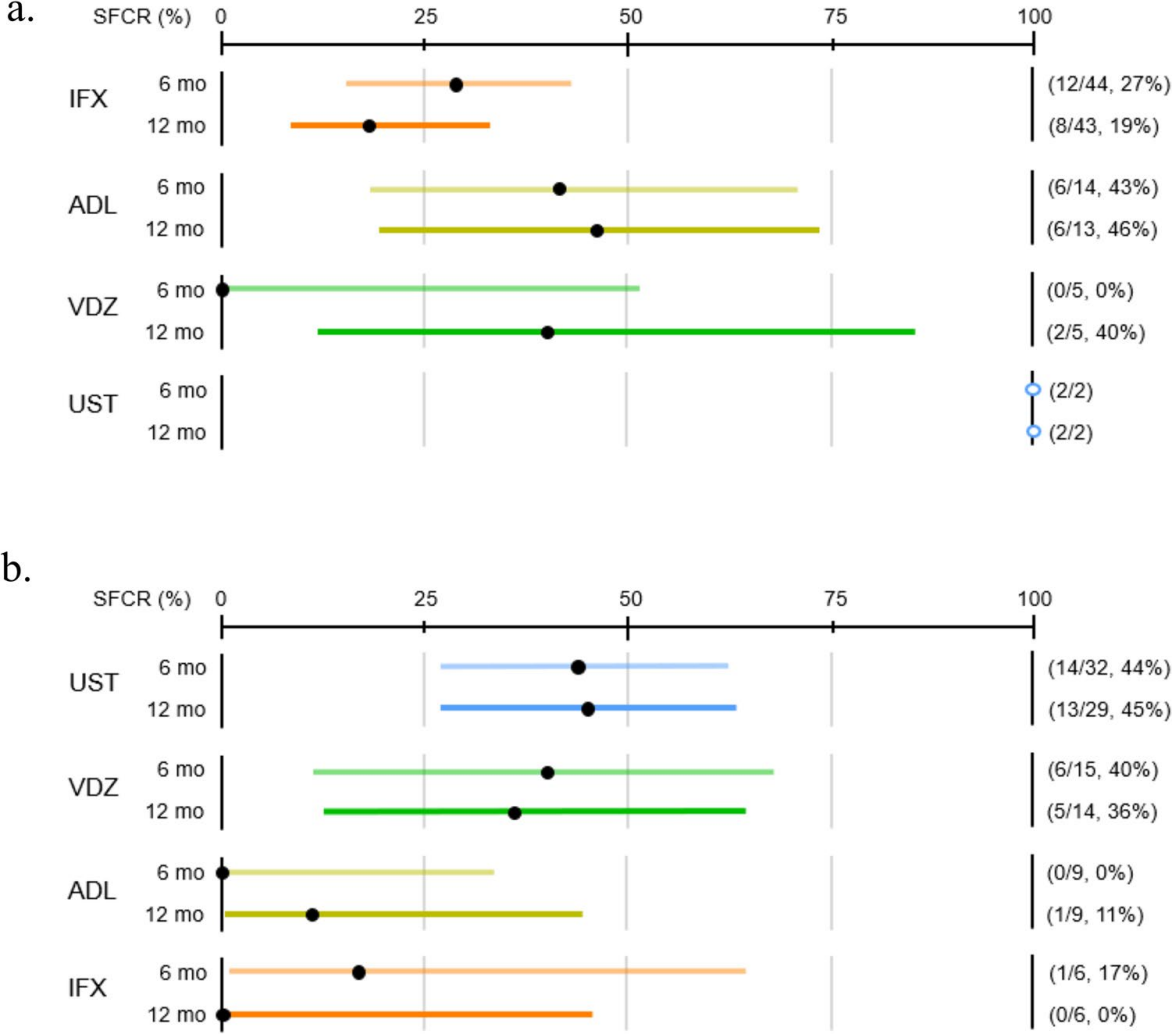
Steroid-free clinical remission (SFCR) at 6 and 12 months. **a** Each biologic used as first-line therapy. **b** Each biologic used as second-line or subsequent therapy. Dots represent SFCR rates; horizontal lines indicate 95% confidence intervals (Clopper–Pearson exact method). Open circles denote groups with fewer than 5 patients (CIs not calculated). *SFCR* steroid-free clinical remission, *IFX* infliximab, *ADL* adalimumab, *VDZ* vedolizumab, *UST* ustekinumab

**Fig. 3:**
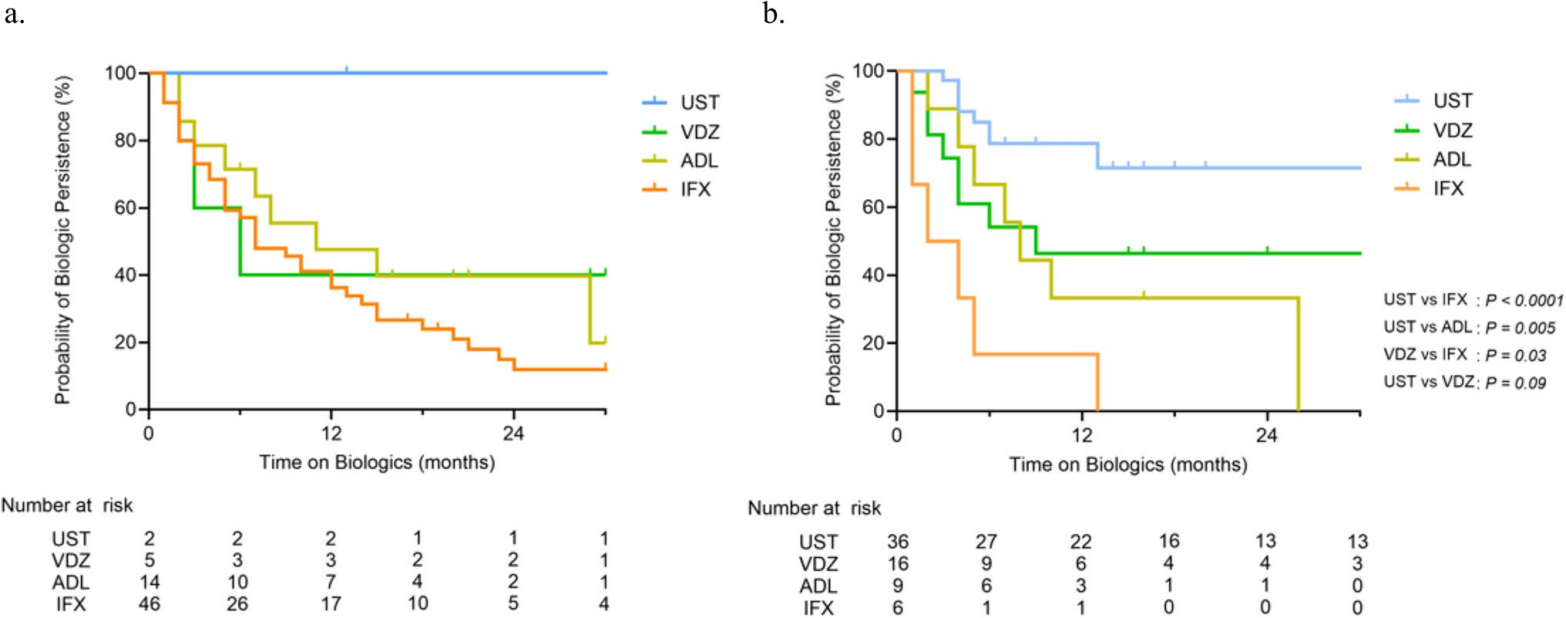
Persistence of biologic therapy. Kaplan–Meier curves showing persistence for each biologic therapy. Orange, blue, yellow, and green bars represent IFX, UST, ADL, and VDZ, respectively. Statistical comparisons were performed using the log-rank (Mantel–Cox) test. **a** First-line therapy, **b** Second-line or subsequent therapy. *IFX* infliximab, *UST* ustekinumab, *ADL* adalimumab, *VDZ* vedolizumab

At 6 and 12 months after biologic initiation, steroid-free clinical remission (SFCR) rates are shown in Fig. 2 and Table 3. Among biologics used as first-line therapy, SFCR at 6 and 12 months was 27%/19% for IFX (*n* = 46), 43%/46% for ADL (*n* = 14), 0%/40% for VDZ (*n* = 5), and 100%/100% for UST (*n* = 2). For biologics used as second-line or subsequent therapies, rates were 44%/45% for UST (*n* = 36), 40%/36% for VDZ (*n* = 16), 0%/11% for ADL (*n* = 9), and 17%/0% for IFX (*n* = 6). Persistence rates are presented in Fig. 3 and Table 3. At 6 and 12 months, persistence was 57%/36% for IFX, 71%/48% for ADL, 40%/40% for VDZ, and 100%/100% for UST among first-line users. For second-line or subsequent use, persistence was 79%/79% for UST, 54%/46% for VDZ, 67%/33% for ADL, and 17%/17% for IFX. Among second-line therapies, persistence appeared higher with UST than with IFX (*P* < 0.0001) and ADL (*P* = 0.005). No significant difference in persistence was observed between UST and VDZ after TNFα inhibitor failure (log-rank *P* = 0.59; Supplementary Fig. 1). In log-rank and Cox proportional hazards analysis (Supplementary Table 2), no significant factors associated with early discontinuation were identified for IFX or ADL, including concomitant immunomodulator use. In contrast, UC/IBDU was linked to earlier discontinuation of UST, and severe disease at initiation to earlier discontinuation of VDZ.

**Table 3:** Treatment outcomes and reasons for discontinuation of each biologic used as (A) first-line and (B) second-line or subsequent therapy

A. First-line therapy				
Outcomes	IFX (*n* = 46)	ADL (*n* = 14)	VDZ (*n* = 5)	UST (*n* = 2)
SFCR rate				
6 months	27%	43%	0%	100%
12 months	19%	46%	40%	100%
Persistence rate				
6 months	57%	71%	40%	100%
12 months	36%	48%	40%	100%

Reasons of discontinuation	IFX (*n* = 38)	ADL (*n* = 10)	VDZ (*n* = 3)	UST (*n* = 0)
Primary failure	11 (29%)	3 (30%)	2 (67%)	0 (0%)
Secondary failure	19 (50%)	6 (60%)	1 (33%)	0 (0%)
Infusion reaction	6 (16%)	1 (10%)	0 (0%)	0 (0%)
Side effects other than IR	1 (2.6%)	0 (0%)	0 (0%)	0 (0%)
Patient’s request	0 (0%)	0 (0%)	0 (0%)	0 (0%)
Others	1 (2.6%)	0 (0%)	0 (0%)	0 (0%)

B. Second-line or subsequent therapy				
Outcomes	UST (*n* = 36)	VDZ (*n* = 16)	ADL (*n* = 9)	IFX (*n* = 6)
SFCR rate				
6 months	44%	40%	0%	17%
12 months	45%	36%	11%	0%
Persistence rate				
6 months	79%	54%	67%	17%
12 months	79%	46%	33%	17%
Reasons of discontinuation	UST (*n* = 13)	VDZ (*n* = 9)	ADL (*n* = 7)	IFX (*n* = 6)
Primary failure	7 (54%)	4 (44%)	3 (43%)	4 (67%)
Secondary failure	3 (23%)	3 (33%)	4 (57%)	2 (33%)
Infusion reaction	0 (0%)	0 (0%)	0 (0%)	0 (0%)
Side effects other than IR	0 (0%)	0 (0%)	0 (0%)	0 (0%)
Patient’s request	2 (15%)	0 (0%)	0 (0%)	0 (0%)
Others	1 (7.7%)	2 (22%)	0 (0%)	0 (0%)

*IFX* infliximab, *ADL* adalimumab, *VDZ* vedolizumab, *UST* Ustekinumab, *SFCR* steroid-free clinical remission, *IR* infusion reaction

### Reasons for discontinuation

Reasons for discontinuation of each biologic agent are summarized in Table 3 and Supplementary Fig. 2. Among biologics used as first-line therapy, secondary loss of response was the most frequent cause of discontinuation, particularly for IFX and ADL. In contrast, for biologics used as second-line or subsequent therapies, primary non-response was more common. Notably, both IFX and UST tended to be discontinued more frequently due to primary failure in patients with UC/IBDU than in those with CD. No discontinuations were reported due to infusion reactions or other adverse events in patients receiving UST or VDZ.

### Doses of biologic agents

Weight-based dose determination was carried out for each biologic at initiation and at final administration (either discontinuation or the end of follow-up), using medians and IQRs (Table 4). The final dose of IFX remained at 8.6 mg/kg (IQR: 6.5–10.2). Evaluation of final dosing intervals showed that intervals were shortened in 57% of IFX-treated patients and 34% of UST-treated patients, while only 5% of those receiving VDZ underwent such dose intensification (Table 4).

**Table 4:** Dose of each biologic agent at initiation and final administration

	IFX (*n* = 52)	UST (*n* = 38)	ADL (*n* = 23)	VDZ (*n* = 21)
Initial administration				
Body weight, median (IQR), kg	13 (10.7–17.6)	14.7 (11–18.5)	14.5 (10.9–17.2)	16.1 (12.4–20)
≥ 30 kg, *n* (%)	0 (0)	0 (0)	0 (0)	0 (0)
Dose, mg/kg, median (IQR)	5.6 (5.3–6.2)	8.6 (6.9–9.9)	40* (40–80)	9.4 (8.1–10.3)
Final administration				
Body weight, median (IQR), kg	15.2 (11.2–19.7)	20 (15.6–22.4)	16.4 (14.0–20.0)	19.7 (13.7–24.0)
≥ 30 kg, *n* (%)	1 (1.9)	2 (5.3)	1 (4.3)	3 (14)
Dose, mg/kg, median (IQR)	8.6 (6.5–10.2)	2.8 (2.3–3.2)	20.0* (20.0–40.0)	8.9 (7.5–10.3)
Final dosing interval^#^, *n* (%)				
≤ 4 weeks	4 (11)	1 (2.6)	–	0
> 4 to 6 weeks	17 (46)	12 (32)	–	1 (5)
> 6 to ≤ 8 weeks	16 (43)	25 (66)	–	19 (95)

*IFX* infliximab, *UST* Ustekinumab, *ADL* adalimumab, *VDZ* vedolizumab, *IQR* interquartile range

*ADL dose is presented in milligrams (mg), not per kilogram

^#^Final dosing interval was not evaluated in patients who discontinued IFX, UST, or VDZ within 2 months of initiation and thus they were excluded from the denominator

For UST and VDZ, comparisons of weight-based dosing at initiation and at final administration between ongoing and discontinuation groups showed no significant differences (Supplementary Fig. 3).

## Discussion

In this multicenter real-world study, we examined the natural history and treatment outcomes of 101 children with VEO-IBD, with a particular focus on clinical utility of biologic agents. First-line IFX showed only 36% persistence at one year, while UST and VDZ, even used as second-line or subsequent therapies, had respective one-year persistence rates of approximately 80 and 50%. No discontinuations due to adverse events—including infusion reactions—were observed in patients treated with UST or VDZ, suggesting favorable long-term tolerability. These findings highlight the therapeutic potential and safety of UST and VDZ in managing VEO-IBD. These agents may play an important role in future treatment strategies once they receive broader approval for pediatric use.

This study also delineated the natural history of VEO-IBD. Both CD and UC demonstrated extensive and severe disease distribution. These characteristics are generally consistent with findings in Western multicenter studies of VEO-IBD. Previous studies report approximately 75–94% of patients with VEO-CD to have colonic involvement, with 50–75% of those with VEO-UC presenting with pancolitis [4, 17]. Our cohort appeared to have somewhat more aggressive disease. In addition, nearly half of our CD cases had perianal involvement. In East Asian cohorts, approximately 35–50% of pediatric and adult CD patients presented with perianal disease [18–20]. Our findings suggest that this phenotype may be frequent in children with VEO-IBD under 6 years of age. Such disease distribution patterns likely have significant impact on treatment strategies. In our cohort, biologic agents were used in two-thirds of patients, with approximately half of cases required switching to a second or third biologic. Previous studies also have reported frequent need for biologic agents in VEO-IBD. For instance, Kerur et al. reported biologic use in 18% of patients within the first year of diagnosis and 41% within 5 years, while Atia et al. noted an overall rate of 35% [17, 21]. These findings reflect a treatment-refractory tendency in VEO-IBD, requiring treatment beyond conventional treatment algorithms. Even early on, more individualized therapeutic approaches may be needed.

When order of treatment was considered in our study, TNF-α inhibitors used as first-line agents showed limited long-term persistence, while UST, even when used as a later-line therapy, demonstrated favorable clinical outcomes over 1 year. In adult IBD, several real-world studies also reported favorable clinical outcomes with UST and VDZ. One large database showed 1-year persistence rates in CD of 91.3% for UST and 87.7% for IFX, while in UC these rates were 84.0% for UST and 62.9% for IFX [22]. Chiu et al. also reported that for patients in whom VDZ had failed, UST showed better treatment persistence than TNF inhibitors [23]. A meta-analysis further indicated that results with VDZ in UC (RR: 1.30) and with UST in CD (RR: 1.15) were more durable than those with TNF inhibitors [24]. In our VEO-IBD cohort, first-line use of IFX showed limited outcomes, with a 1-year SFCR rate of 19% and a persistence rate of 36%. In contrast, UST achieved sustained use even when introduced as a second-line or subsequent therapy, with approximately 80% of patients remaining on treatment at 1 year—suggesting a larger difference between agents than previously reported in adult populations. Poor persistence with IFX in VEO-IBD has also been described in earlier pediatric studies. In contrast to the high persistence rates reported in the REACH trial (93% at 1 year), later studies focusing on VEO-IBD have shown much lower IFX persistence (36% at 1 year) and SFCR persistence as low as 9% [25, 26, 27]. This difference may be related to differing pharmacokinetics of IFX in younger children, including higher drug clearance, reduced trough levels, and shorter half-life [28, 29]. Recent pediatric pharmacokinetic studies have also indicated that similar considerations apply to other biologics such as ADL, and possibly UST, which may require dosing optimization (e.g., intensification or interval shortening) due to altered pharmacokinetics in children [30–32]. However, in our cohort, only one-third of patients receiving UST underwent interval shortening. These findings suggest that UST appeared to be effective under standard dosing conditions, although further optimization, such as therapeutic drug monitoring and proactive interval adjustment, may warrant consideration in future studies.

In contrast, VDZ showed a 1-year persistence rate of 46%, which was favorable considering that most patients received it as a second-line or subsequent therapy. Similar findings were reported in pediatric cohorts including the VEDOKIDS study, which showed a 1-year persistence rate of 47% among previously treated children [33]. Additionally, in our cohort, VDZ doses administered were slightly lower than those reported in prior studies, but only 5% of patients required interval shortening. Even so, recent evidence suggests that higher doses or shortened intervals may help to sustain VDZ efficacy in refractory cases [34, 35]. Possibly underutilization of optimization strategies contributed to lower persistence rates in our study.

No patients in our cohort discontinued UST or VDZ due to adverse events during the study period. Although data concerning use of UST and VDZ in VEO-IBD remain limited, prior pediatric IBD studies reported favorable safety profiles for both agents [33, 36–38]. Our findings are consistent with those reports and support the notion that UST and VDZ appear safe in young children with VEO-IBD.

This study has several limitations. First, case numbers were relatively small, although this remains one of the larger multicenter cohorts of VEO-IBD reported to date. Second, this was a retrospective observational study in which treatment decisions—including drug discontinuation, dosing, and interval adjustments—were made at the discretion of treating physicians. Therapeutic adjustments were not standardized, and timing and definitions of dose escalation or interval shortening varied among patients. For IFX, these modifications were not guided by trough concentration monitoring, which are difficult to obtain in Japan due to insurance constraints. As suggested by Stallard et al., future comparative studies using body surface area-based dosing may be considered in [39]. Third, our study population included both biologic-naïve patients and others who received second-line or subsequent therapy following biologic failure, which could have introduced heterogeneity in treatment outcomes. Future prospective studies, ideally in biologic-naive patients, are needed to more rigorously evaluate the efficacy of UST and VDZ in VEO-IBD.

## Conclusion

This multicenter retrospective study found UST and VDZ to be effective and safe options for VEO-IBD, even when used as second-line or subsequent therapies. Future prospective studies addressing therapeutic optimization of drug concentrations are warranted.

## Supplementary Information

Below is the link to the electronic supplementary material.

Supplementary tables and figures providing additional analyses of biologic therapy outcomes.

## Abbreviations

VEO-IBD: Very-early-onset inflammatory bowel disease
UST: Ustekinumab
VDZ: Vedolizumab
IFX: Infliximab
ADL: Adalimumab
SFCR: Steroid-free clinical remission
IBD: Inflammatory bowel disease
UC: Ulcerative colitis
CD: Crohn’s disease
IBDU: IBD-unclassified
TNF: Tumor necrosis factor-alpha
PUCAI: Pediatric ulcerative colitis activity index
wPCDAI: Weighted pediatric Crohn’s disease activity index
IQR: Interquartile ranges

## References

[1] Kuenzig ME, Fung SG, Marderfeld L, Mak JWY, Kaplan GG, Ng SC, et al. Twenty-first century trends in the global epidemiology of pediatric-onset inflammatory bowel disease: systematic review. Gastroenterology. 2022;162:1147-1159.e4. 34995526 10.1053/j.gastro.2021.12.282

[2] Stulman MY, Asayag N, Focht G, Brufman I, Cahan A, Ledderman N, et al. Epidemiology of inflammatory bowel diseases in Israel: a nationwide Epi-Israeli IBD research nucleus study. Inflamm Bowel Dis. 2021;27:1784–94. 33438721 10.1093/ibd/izaa341

[3] Crowley E, Warner N, Pan J, Khalouei S, Elkadri A, Fiedler K, et al. Prevalence and clinical features of inflammatory bowel diseases associated with monogenic variants, identified by whole-exome sequencing in 1000 children at a single center. Gastroenterology. 2020;158:2208–20. 32084423 10.1053/j.gastro.2020.02.023 PMC7283012

[4] Guz-Mark A, Aloi M, Scarallo L, Bramuzzo M, Escher JC, Alvisi P, et al. Infantile and very early onset inflammatory bowel disease: a multicenter study. Pediatrics. 2024;154:e2023064546. 39015095 10.1542/peds.2023-064546

[5] Cucinotta U, Arrigo S, Dipasquale V, Gramaglia SMC, Laganà F, Romano C, et al. Clinical course of very early-onset inflammatory bowel disease. J Pediatr Gastroenterol Nutr. 2023;76:590–5. 36754082 10.1097/MPG.0000000000003730

[6] Lee WS, Chew KS, Huang JG, Tanpowpong P, Mercado KSC, Reodica A, et al. Disease phenotypic and outcome of very-early onset inflammatory bowel disease in Asian children: an understudied population. Front Pediatr. 2025;13:1487253. 40051907 10.3389/fped.2025.1487253 PMC11882516

[7] Kurowski JA, Milinovich A, Ji X, Bauman J, Sugano D, Kattan MW, et al. Differences in biologic utilization and surgery rates in pediatric and adult Crohn’s disease: results from a large electronic medical record-derived cohort. Inflamm Bowel Dis. 2021;27:1035–44. 32914165 10.1093/ibd/izaa239

[8] Kaplan JL, Liu C, King EC, Bass JA, Patel AS, Tung J, et al. Use, durability, and risks for discontinuation of initial and subsequent biologics in a large pediatric-onset IBD cohort. J Pediatr Gastroenterol Nutr. 2023;76:566–75. 36804501 10.1097/MPG.0000000000003734 PMC10097486

[9] Urushiyama M, Tarasawa K, Moroi R, Iwaki H, Hoshi Y, Nagai H, et al. Evolving trends in pediatric inflammatory bowel disease management in Japan: a decade of nationwide data. JGH Open. 2025;9:e70175. 40375856 10.1002/jgh3.70175 PMC12078194

[10] Collen LV, Mitsialis V, Kim DY, Bresnahan M, Yang J, Tuthill M, et al. Efficacy and safety of anti-tumor necrosis factor alpha in very early onset inflammatory bowel disease. Inflamm Bowel Dis. 2024;30:1443–53. 37847820 10.1093/ibd/izad196 PMC11369069

[11] Weintraub Y, Collen LV, Hussey S, Mitrova K, Machta JS, Kang B, et al. Effectiveness and safety of adalimumab in patients with very early-onset inflammatory bowel disease: a retrospective study on behalf of the Porto Inflammatory Bowel Disease Working Group of European Society for Pediatric Gastroenterology Hepatology and Nutrition. Inflamm Bowel Dis. 2025. 10.1093/ibd/izae302. 39813158 10.1093/ibd/izae302

[12] Nambu R, Warner N, Mulder DJ, Kotlarz D, McGovern DPB, Cho J, et al. A systematic review of monogenic inflammatory bowel disease. Clin Gastroenterol Hepatol. 2022;20:e653–63. 33746097 10.1016/j.cgh.2021.03.021 PMC8448782

[13] Levine A, Koletzko S, Turner D, Escher JC, Cucchiara S, de Ridder L, et al. ESPGHAN revised porto criteria for the diagnosis of inflammatory bowel disease in children and adolescents. J Pediatr Gastroenterol Nutr. 2014;58:795–806. 24231644 10.1097/MPG.0000000000000239

[14] Levine A, Griffiths A, Markowitz J, Wilson DC, Turner D, Russell RK, et al. Pediatric modification of the Montreal classification for inflammatory bowel disease: the Paris classification. Inflamm Bowel Dis. 2011;17:1314–21. 21560194 10.1002/ibd.21493

[15] Turner D, Griffiths AM, Walters TD, Seah T, Markowitz J, Pfefferkorn M, et al. Mathematical weighting of the pediatric Crohn’s disease activity index (PCDAI) and comparison with its other short versions. Inflamm Bowel Dis. 2012;18:55–62. 21351206 10.1002/ibd.21649

[16] Turner D, Otley AR, Mack D, Hyams J, de Bruijne J, Uusoue K, et al. Development, validation, and evaluation of a pediatric ulcerative colitis activity index: a prospective multicenter study. Gastroenterology. 2007;133:423–32. 17681163 10.1053/j.gastro.2007.05.029

[17] Kerur B, Benchimol EI, Fiedler K, Stahl M, Hyams J, Stephens M, et al. Natural history of very early onset inflammatory bowel disease in North America: a retrospective cohort study. Inflamm Bowel Dis. 2021;27:295–302. 32386060 10.1093/ibd/izaa080 PMC8177809

[18] Arai K, Kunisaki R, Kakuta F, Hagiwara SI, Murakoshi T, Yanagi T, et al. Phenotypic characteristics of pediatric inflammatory bowel disease in Japan: results from a multicenter registry. Intest Res. 2020;18:412–20. 32806870 10.5217/ir.2019.00130 PMC7609396

[19] Lee HA, Suk JY, Choi SY, Kim ER, Kim YH, Lee CK, et al. Characteristics of pediatric inflammatory bowel disease in Korea: comparison with EUROKIDS data. Gut Liver. 2015;9:756–60. 25963086 10.5009/gnl14338 PMC4625705

[20] Yamamoto T, Nakase H, Watanabe K, Shinzaki S, Takatsu N, Fujii T, et al. Diagnosis and clinical features of perianal lesions in newly diagnosed Crohn’s disease: subgroup analysis from inception Cohort Registry Study of Patients with Crohn’s Disease (iCREST-CD). J Crohns Colitis. 2023;17:1193–206. 36869815 10.1093/ecco-jcc/jjad038 PMC10441562

[21] Atia O, Benchimol EI, Ledderman N, Greenfeld S, Kariv R, Weisband YL, et al. Incidence, management, and outcomes of very early onset inflammatory bowel diseases and infantile-onset disease: an Epi-IIRN study. Clin Gastroenterol Hepatol. 2023;21:2639-2648.e6. 36336312 10.1016/j.cgh.2022.10.026

[22] Matsuoka K, Nakajo K, Kawamura S, Zhang Y, Chung H, Wahking B, et al. Persistence of advanced therapies in patients with inflammatory bowel disease: retrospective cohort study using a large healthcare claims database in Japan. Intest Res. 2025. 10.5217/ir.2024.00118. 39701922 10.5217/ir.2024.00118 PMC12332283

[23] Chiu HY, Kuo CJ, Lai MW, Wu RC, Chen CM, Chiu CT, et al. Superior persistence of ustekinumab compared to anti-TNF in vedolizumab-experienced inflammatory bowel diseases patients: a real-world cohort study. BMC Gastroenterol. 2024;24:483. 39741232 10.1186/s12876-024-03577-1 PMC11686934

[24] Yiu TH, Ko Y, Pudipeddi A, Natale P, Leong RW. Meta-analysis: persistence of advanced therapies in the treatment of inflammatory bowel disease. Aliment Pharmacol Ther. 2024;59:1312–34. 38651771 10.1111/apt.18006

[25] Hyams J, Crandall W, Kugathasan S, Griffiths A, Olson A, Johanns J, et al. Induction and maintenance infliximab therapy for the treatment of moderate-to-severe Crohn’s disease in children. Gastroenterology. 2007;132:863–73. 17324398 10.1053/j.gastro.2006.12.003

[26] Bramuzzo M, Arrigo S, Romano C, Filardi MC, Lionetti P, Agrusti A, et al. Efficacy and safety of infliximab in very early onset inflammatory bowel disease: a national comparative retrospective study. United Eur Gastroenterol J. 2019;7:759–66. 10.1177/2050640619847592 PMC6620882 31316780

[27] Kelsen JR, Sullivan KE, Rabizadeh S, Singh N, Snapper S, Elkadri A, et al. North American Society for Pediatric Gastroenterology, Hepatology, and Nutrition position paper on the evaluation and management for patients with very early-onset inflammatory bowel disease. J Pediatr Gastroenterol Nutr. 2020;70:389–403. 32079889 10.1097/MPG.0000000000002567 PMC12024488

[28] Jongsma MME, Winter DA, Huynh HQ, Norsa L, Hussey S, Kolho KL, et al. Infliximab in young paediatric IBD patients: it is all about the dosing. Eur J Pediatr. 2020;179:1935–44. 32813123 10.1007/s00431-020-03750-0 PMC7666662

[29] deBruyn JCC, Jacobson K, El-Matary W, Wine E, Carroll MW, Goedhart C, et al. Early serum infliximab levels in pediatric ulcerative colitis. Front Pediatr. 2021;9:668978. 34395336 10.3389/fped.2021.668978 PMC8358797

[30] Croft NM, Faubion WA Jr, Kugathasan S, Kierkus J, Ruemmele FM, Shimizu T, et al. Efficacy and safety of adalimumab in paediatric patients with moderate-to-severe ulcerative colitis (ENVISION I): a randomised, controlled, phase 3 study. Lancet Gastroenterol Hepatol. 2021;6:616–27. 34153231 10.1016/S2468-1253(21)00142-4

[31] Rosh JR, Turner D, Griffiths A, Cohen SA, Jacobstein D, Adedokun OJ, et al. Ustekinumab in paediatric patients with moderately to severely active Crohn’s disease: pharmacokinetics, safety, and efficacy results from UniStar, a phase 1 study. J Crohns Colitis. 2021;15:1931–42. 34037715 10.1093/ecco-jcc/jjab089 PMC8575045

[32] Turner D, Rosh JR, Cohen SA, Griffiths AM, Hyams JS, Kierkuś J, et al. Ustekinumab in paediatric patients with moderately to severely active Crohn’s disease: UniStar study long-term extension results. J Pediatr Gastroenterol Nutr. 2024;79:315–24. 38801079 10.1002/jpn3.12252

[33] Atia O, Shavit-Brunschwig Z, Mould DR, Stein R, Matar M, Aloi M, et al. Outcomes, dosing, and predictors of vedolizumab treatment in children with inflammatory bowel disease (VEDOKIDS): a prospective, multicentre cohort study. Lancet Gastroenterol Hepatol. 2023;8:31–42. 36306803 10.1016/S2468-1253(22)00307-7

[34] Peyrin-Biroulet L, Danese S, Argollo M, Pouillon L, Peppas S, Gonzalez-Lorenzo M, et al. Loss of response to vedolizumab and ability of dose intensification to restore response in patients with Crohn’s disease or ulcerative colitis: a systematic review and meta-analysis. Clin Gastroenterol Hepatol. 2019;17:838-846.e2. 29935327 10.1016/j.cgh.2018.06.026

[35] Vaughn BP, Yarur AJ, Graziano E, Campbell JP, Bhattacharya A, Lee JY, et al. Vedolizumab serum trough concentrations and response to dose escalation in inflammatory bowel disease. J Clin Med. 2020;9:3142. 32998473 10.3390/jcm9103142 PMC7601452

[36] Singh N, Rabizadeh S, Jossen J, Pittman N, Check M, Hashemi G, et al. Multi-center experience of vedolizumab effectiveness in pediatric inflammatory bowel disease. Inflamm Bowel Dis. 2016;22:2121–6. 27542130 10.1097/MIB.0000000000000865

[37] Rébus S, Coopman S, Djeddi D, Vanrenterghem A, Dupont C, Lacotte E, et al. Efficacy of vedolizumab and ustekinumab in pediatric-onset inflammatory bowel disease: a real-world multicenter study. J Pediatr Gastroenterol Nutr. 2025;80:113–23. 39415517 10.1002/jpn3.12384

[38] Cohen S, Rolandsdotter H, Kolho KL, Turner D, Tzivinikos C, Bramuzzo M, et al. Effectiveness and safety of ustekinumab in pediatric ulcerative colitis: a multi-center retrospective study from the Pediatric IBD Porto Group of ESPGHAN. Paediatr Drugs. 2024;26:609–17. 38780740 10.1007/s40272-024-00631-z PMC11335845

[39] Stallard L, Frost K, Frost N, Scarallo L, Benchimol EI, Walters TD, et al. Body surface area-based dosing of infliximab is superior to standard weight-based dosing in children with very early onset inflammatory bowel disease. Gastro Hep Adv. 2023;3:215–20. 39129953 10.1016/j.gastha.2023.11.004 PMC11308830

