# Supplementary material for: Real-world outcomes of ustekinumab, vedolizumab, and tumor necrosis factor inhibitors in very-early-onset inflammatory bowel disease: a multi-center cohort study
Source: J Gastroenterol. 2026 Jan 3;61(4):398–409. doi: 10.1007/s00535-025-02334-9 (PMC13048906; doi:10.1007/s00535-025-02334-9)
Supplement: Supplementary file 1 — Supplementary tables and figures providing additional analyses of biologic therapy outcomes. [file 535_2025_2334_MOESM1_ESM.docx]

**Supplementary Table, Figures and Figure Legends**

**Real-world Outcomes of Ustekinumab, Vedolizumab, and Tumor Necrosis Factor Inhibitors in Very Early Onset Inflammatory Bowel Disease:**

**A Multicenter Cohort Study**

**Supplementary Tables　 Page 2 - 4**

**Supplementary Figures and Figure Legends Page 5 - 9**

**Supplementary Table 1.** Baseline patient characteristics stratified by disease type (Crohn’s disease vs UC/IBD-U).

|  |  | CD (n=40) | UC/IBDU (n=61) |
| --- | --- | --- | --- |
| Male, n (%) | | 22 | 34 |
| Age at diagnosis, median (IQR), years | | 3.9 (2.4–5.3) | 3.6 (2.8–5.3) |
| Family history^＃^, n (%) | | 2 | 4 |
| Observation period, median (IQR), years | | 3.7 (2.2–4.9) | 3.8 (2.6–5.4) |
| Severe disease at diagnosis, n (%)  (CD: wPCDAI > 57.5, UC/IBDU: PUCAI ≥ 65) | | 13 | 10 |
| Laboratory data,  median (IQR) | Hgb (mg/dL) | 10.9 (8.8–12.0) | 11.0 (9.7–11.9) |
|  | Alb (g/dL） | 3.4 (2.8–3.9) | 3.7 (3.1–4.3) |
|  | CRP (mg/dL) | 0.41 (0. 11–5.27） | 0.19 (0.06–0.66） |
|  | ESR (mm/1h) | 35 (19–55) | 16 (8–21) |
| Location of CD involvement,  n (%) | L1 | 3 (8) |  |
|  | L2 | 24 (60) |  |
|  | L3 | 12 (30) |  |
|  | L4a | 19 (48) |  |
|  | L4b | 10 (25) |  |
|  | B1 | 34 (83) |  |
|  | B2 | 3 (8) |  |
|  | B3 | 1 (3) |  |
|  | p | 18 (45) |  |
| Location of UC/IBD-U involvement,　n (%) | E2 |  | 1 (1.6) |
|  | E3 |  | 6 (10) |
|  | E4 |  | 54 (89) |
| Non-biologic medication, | 5-ASA | 26 (65) | 55 (90) |
| n (%) Systemic corticosteroid | | 22 (55) | 49 (80) |
|  | IM | 25 (63) | 41 (67) |
|  | Tac/Cys | 0 (0) | 7 (11) |
| Number of biologic agents,  n (%) | ≥ 1 | 31 (78) | 36 (59) |
|  | ≥ 2 | 20 (50) | 25 (41) |
|  | ≥ 3 | 9 (22) | 14 (23) |
| Biologic agents used,  n (%) | IFX | 26 (65) | 26 (43) |
|  | UST | 18 (45) | 20 (33) |
|  | ADL | 12 (30) | 11 (18) |
|  | VDZ | 4 (10) | 17 (28) |
|  | TOF | 0 (0) | 3 (4.9) |
|  | UPA | 0 (0) | 3 (4.9) |
|  | RKZ | 1 (2.5) | 2 (3.3) |
|  | GLM | 0 (0) | 2 (3.3) |
|  | MKZ | 0 (0) | 1 (1.6) |
| Surgery, | Colectomy | 1 (2.5) | 5 (8.2) |
| n (%) | Ileostomy/colostomy | 1 (2.5) | 6 (9.8) |
|  | Seton insertion | 6 (15) | 0 (0) |

^#^Within second-degree relatives

CD, Crohn’s disease; UC, ulcerative colitis; IBD-U, inflammatory bowel disease-unclassified; IQR, interquartile range; wPCDAI, weighted Pediatric Crohn’s Disease Activity Index; PUCAI, Pediatric Ulcerative Colitis Activity Index; Hgb, hemoglobin; ALB, albumin; CRP, C-reactive protein; ESR, erythrocyte sedimentation rate; L, location; B, behavior; p, perianal disease; E, extent; ASA, aminosalicylic acid; IM, immunomodulators; TAC/Cys, tacrolimus/cyclosporine; IFX, infliximab; UST, ustekinumab; ADL, adalimumab; VDZ, vedolizumab; TOF, tofacitinib; UPA, upadacitinib; RKZ, risankizumab; GLM, golimumab; MKZ, mirikizumab.

**Supplementary Table 2. Log-rank and Cox proportional hazards analyses for persistence of each biologic therapy (a. IFX, b. ADL, c. UST, d. VDZ).**

1. **IFX**

|  | n (%) | Univariate | | |  | Multivariate | | |
| --- | --- | --- | --- | --- | --- | --- | --- | --- |
|  |  | HR | 95%CI | P |  | HR | 95%CI | P |
| Disease type: UC/IBDU (vs CD) | 26 (50) | 1.71 | 0.93–3.14 | 0.08 |  | 1.65 | 0.87–3.14 | 0.12 |
| Severe disease at initiation^＃^ | 14 (27) | 0.99 | 0.50–1.98 | 0.99 |  | 0.97 | 0.47–1.99 | 0.92 |
| Without immunomodulator use | 18 (35) | 0.93 | 0.49–1.77 | 0.83 |  | 0.99 | 0.50–1.98 | 0.98 |
| Initiation >6 months after diagnosis | 22 (43) | 0.99 | 0.54–1.83 | 0.99 |  | 0.99 | 0.52–1.92 | 0.99 |

1. **ADL**

|  | n (%) | Univariate | | |
| --- | --- | --- | --- | --- |
|  |  | HR | 95%CI | P |
| Disease type: UC/IBDU (vs CD) | 11 (23) | 0.99 | 0.37–2.66 | 0.98 |
| Without immunomodulator use | 12 (52) | 0.97 | 0.36–2.59 | 0.95 |
| Initiation >6 months after diagnosis | 12 (52) | 2.24 | 0.74–6.83 | 0.16 |

1. **UST**

|  | n (%) | Univariate | | |
| --- | --- | --- | --- | --- |
|  |  | HR | 95%CI | P |
| **Disease type: UC/IBDU (vs CD)** | **20 (53)** | **4.83** | **1.02–22.9** | **0.047** |
| Severe disease at initiation^＃^ | 7 (38) | 2.18 | 0.61–7.73 | 0.23 |
| Initiation >6 months after diagnosis | 28 (74) | 0.67 | 0.19–2.35 | 0.54 |

1. **VDZ**

|  | n (%) | Univariate | | |
| --- | --- | --- | --- | --- |
|  |  | HR | 95%CI | P |
| Disease type: UC/IBDU (vs CD) | 17 (81) | 0.78 | 0.17–3.61 | 0.75 |
| **Severe disease at initiation**^＃^ | **5 (24)** | **5.98** | **1.43–25.0** | **0.01** |
| Initiation >6 months after diagnosis | 2 (4.8) | 0.70 | 0.09–5.48 | 0.73 |

# Severe disease at initiation was defined as wPCDAI>57.5 (CD) or PUCAI ≥65(UC/IBDU). Univariable analysis: log-rank (Mantel–Cox) test; multivariable analysis: Cox proportional hazards model. Outcome: time to discontinuation or switch. HR >1 indicates shorter persistence.

IFX, infliximab; ADL, adalimumab; UST, ustekinumab; VDZ, vedolizumab; UC, ulcerative colitis; IBD-U, inflammatory bowel disease-unclassified; CD, Crohn’s disease; wPCDAI, weighted Pediatric Crohn’s Disease Activity Index; PUCAI, Pediatric Ulcerative Colitis Activity Index

**Supplementary Figure 1. Persistence of ustekinumab and vedolizumab following anti-TNFα failure**

**
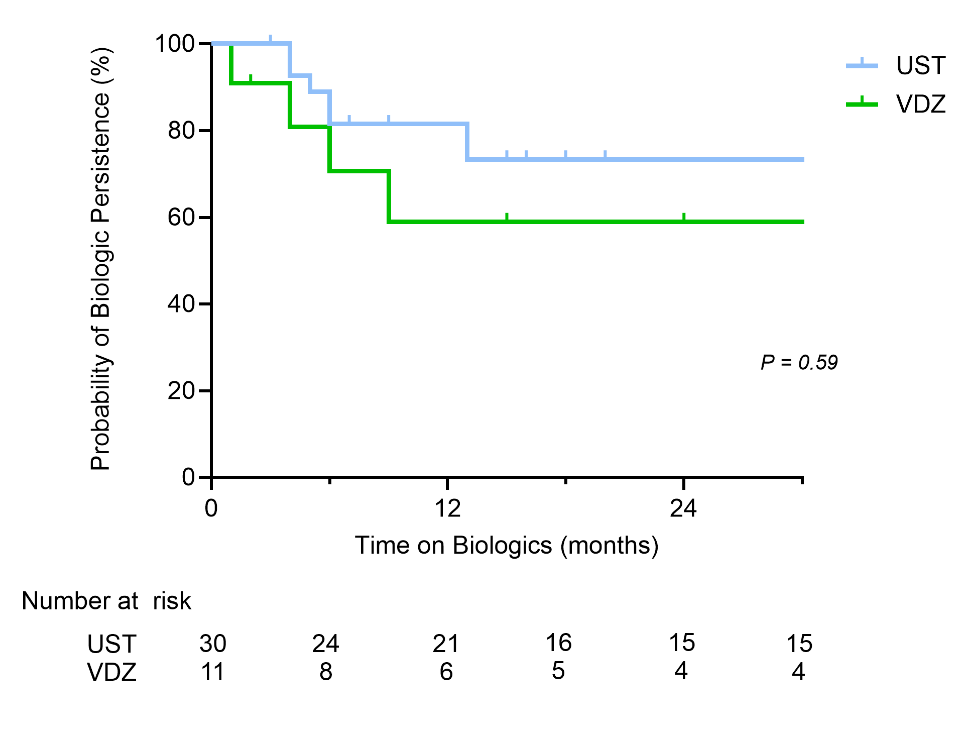
**

Kaplan–Meier curves showing persistence of ustekinumab (UST, blue) and vedolizumab (VDZ, green) initiated immediately after anti-TNFα failure. Persistence was defined as continued use of the same biologic without discontinuation or switching. No significant difference in persistence was observed between the two groups (log-rank test, *P* = n.s.).
UST, ustekinumab; VDZ, vedolizumab.

**Supplementary Figure 2. Reasons for discontinuation of each biologic agent (a. all patients, b. CD, c. UC/IBDU).**

1. All patients


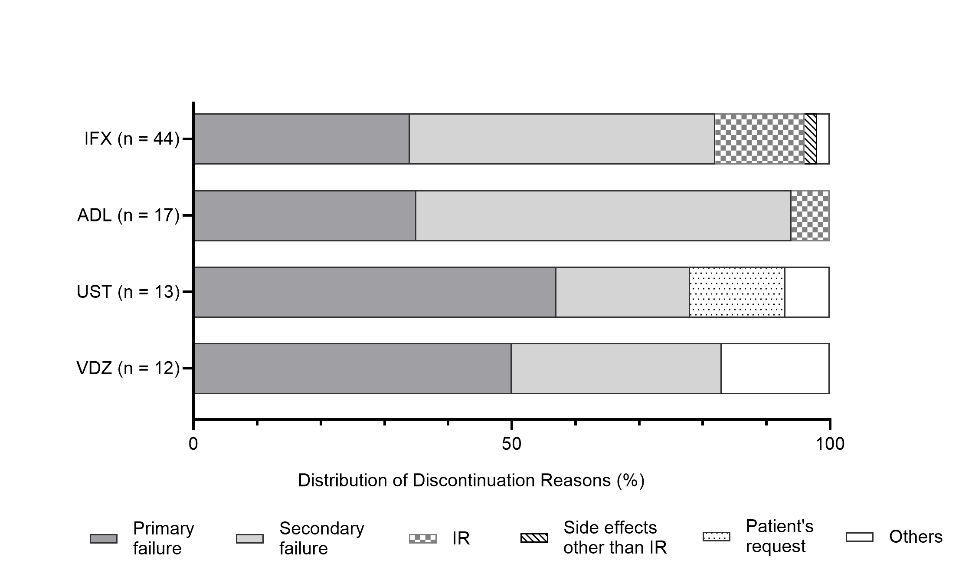


1. CD


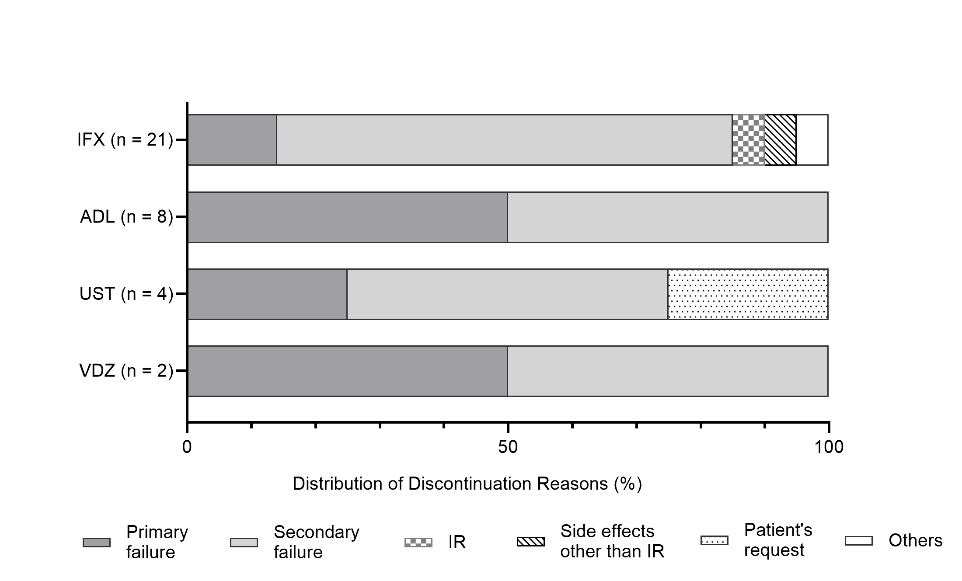


1. UC/IBDU


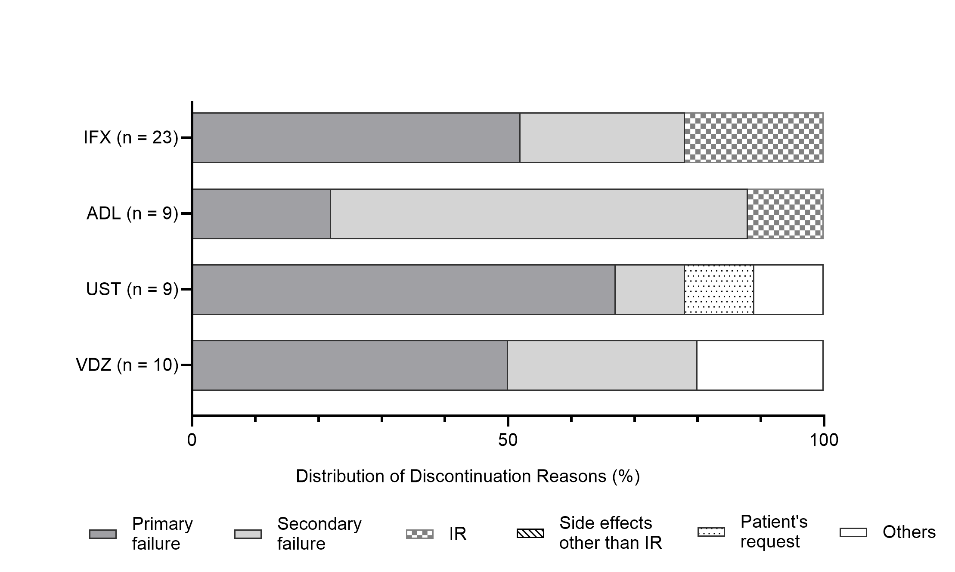


Stacked horizontal bars represent the distribution of discontinuation reasons for IFX, UST, ADL, and VDZ. CD, Crohn’s disease; UC, ulcerative colitis; IBD-U, inflammatory bowel disease-unclassified; IFX, infliximab; UST, ustekinumab; ADL, adalimumab; VDZ, vedolizumab; IR, infusion reaction.

**Supplementary Figure 3. Comparison of ustekinumab (UST) and vedolizumab (VDZ) doses between ongoing and discontinuation groups.**

**A 　UST - Initial** 　　　　　　　　　**B**　 **UST – Final administered Dose**

**
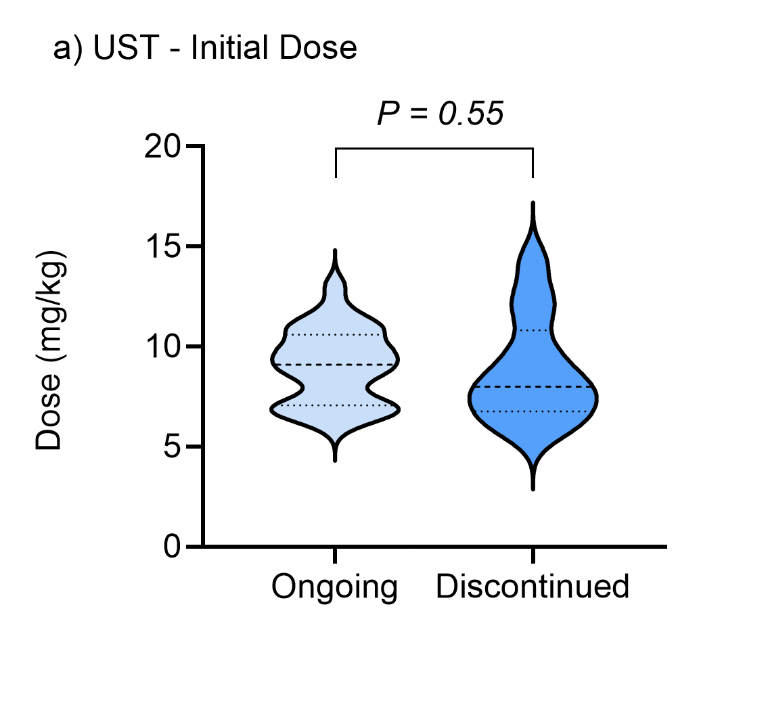
** **
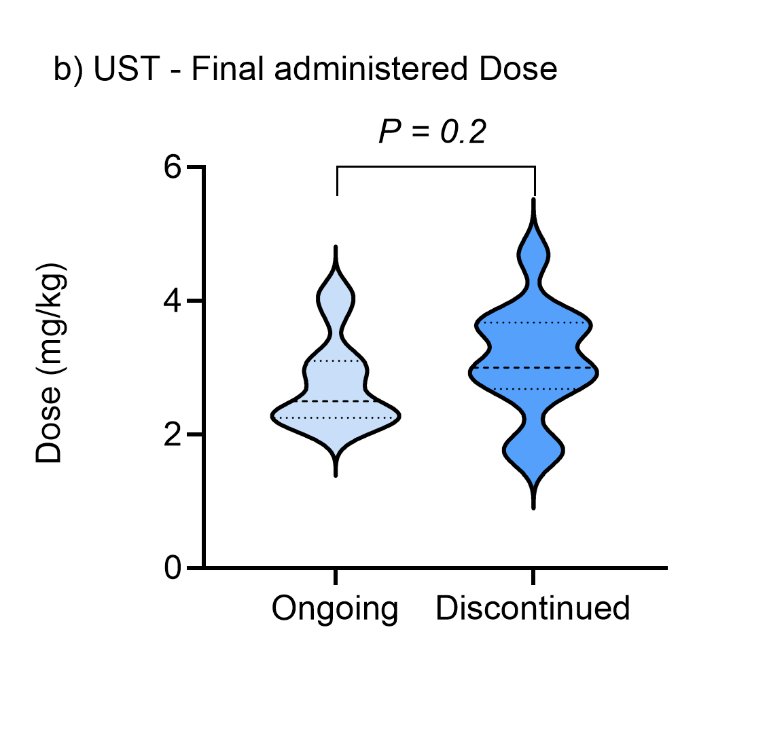
**

**C　VDZ - Initial 　　　　　　　　　　　　D　 VDZ – Final administered Dose**


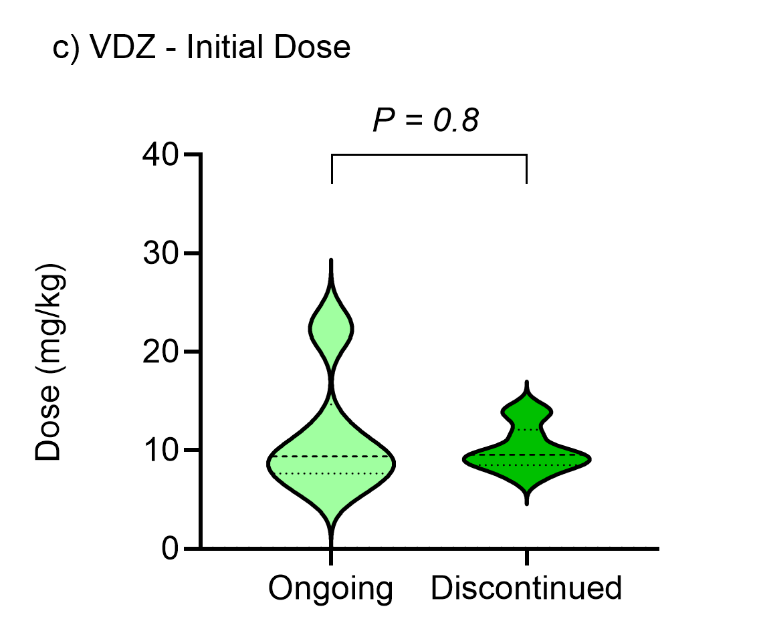

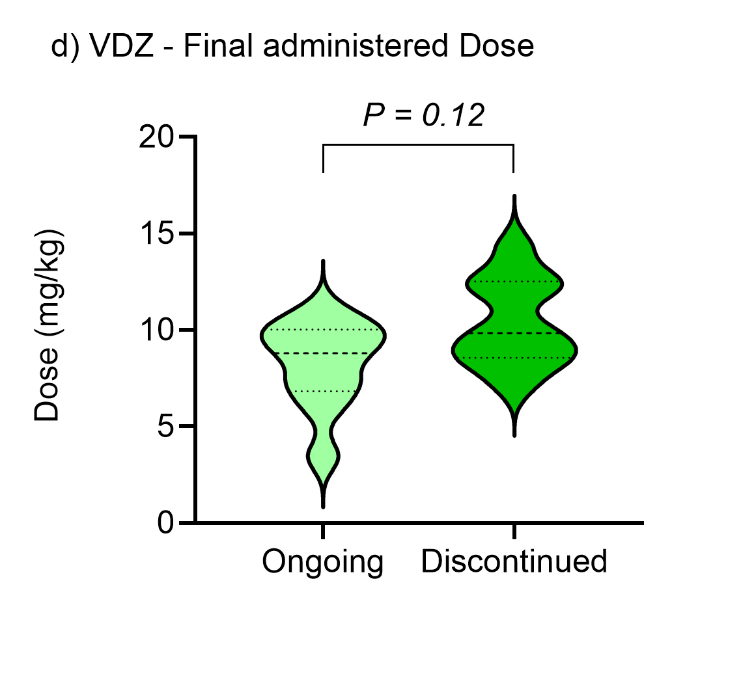


**A** Initial weight-based UST dose. **B** Final weight-based UST dose (defined as the last dose before discontinuation or at the end of the observation period).

**C** Initial weight-based VDZ dose. **D** Final weight-based VDZ dose (defined as the last dose before discontinuation or at the end of the observation period).

Statistical comparisons were performed using the Mann–Whitney U test.

UST, ustekinumab; VDZ, vedolizumab
